# Messaging Impacts Public Perspectives Towards Fur Farming in the Northeastern United States

**DOI:** 10.3390/ani15213158

**Published:** 2025-10-30

**Authors:** Lori R. Kogan, Rebecca Niemiec, Andrew Mertens

**Affiliations:** 1 Clinical Sciences Department, Colorado State University, Fort Collins, CO 80523, USA; 2 Animal-Human Policy Center, Colorado State University, Fort Collins, CO 80523, USA; 3 Department of Biostatistics, University of California, Berkeley, CA 94720, USA

**Keywords:** fur farming, public attitude, policy support, message framing, animal welfare, environmental sustainability, public health, social norm, consumer behavior

## Abstract

**Simple Summary:**

This study examines public attitudes toward fur farming and fur sales bans across four northeastern U.S. states—Connecticut, Massachusetts, New Jersey, and New York—and investigates how different message framings influence support for policy restrictions. Using survey data from over 2000 adults, we found that a majority supported bans on fur sales, particularly when messages emphasized animal welfare, environmental harm, public health risks, or shifting social norms. Political affiliation moderated responses, with independents showing the greatest shift in attitudes. These findings offer practical guidance for policymakers and advocates aiming to design effective communication strategies around fur-related legislation.

**Abstract:**

Animal fur has long symbolized luxury and social status, but growing concerns about animal welfare, environmental harm, and zoonotic disease risks have prompted global reforms, with over 22 countries banning fur production. In the United States, however, public attitudes toward fur farming and sales bans remain underexplored. This study surveyed 2014 adults from Connecticut, Massachusetts, New Jersey, and New York to assess views on fur farming, acceptability, and support for state-level bans, as well as the influence of message framing. Participants were randomly assigned to one of six message conditions (animal welfare, environmental, public health, economic, faux fur alternatives, or social norms) or a control group. Most respondents supported bans on fur sales and fur from commercial farms (approximately 65% weighted). Messages highlighting animal welfare, environmental impacts, public health, and social norms significantly increased support, while economic and faux fur messages did not. Political affiliation moderated these effects, with independents most responsive. Beliefs about cruelty, environmental harm, and zoonotic risks predicted support, whereas conservatism, opposition to regulation, and consumer rights beliefs predicted opposition. Overall, appeals to ethics, sustainability, and social change appear most effective for advancing fur-related policy initiatives.

## 1. Introduction

Clothing and other products made from animal fur have historically been associated with wealth, luxury, and social distinction, symbolizing both economic privilege and cultural prestige [1]. Yet, activism by animal advocates, shifting consumer preferences, and changing social norms have reduced the demand for and production of fur, particularly in Western markets [2,3]. Internationally, momentum for reform has accelerated with at least 22 countries—including the United Kingdom, Austria, and Israel—having implemented bans on fur farming or sales [4,5]. Within the UK, for example, survey research found that more than 90% of residents express concern for the welfare of fur-farmed species, and a large majority support a total ban on fur imports and sales [6]. Similarly, in the United States, polling suggests that nearly three-quarters of likely voters express concern about fur, with a majority supporting bans on fur sales [7]. Despite these shifts, approximately 100 million animals are still killed annually for the global fur trade [6,8]. These animals are primarily American mink (*Neogale vision*), red and Arctic foxes (*Vulpes vulpes*; *Vulpes lagopus*), raccoon dogs (*Nyctereutes procyonoides*), and chinchillas (*Chinchilla lanigera*); all of which have solitary, semi-aquatic, or wide-ranging natural environments which are fundamentally incompatible with life in cages. As a result, current housing and management systems for these species cause serious welfare concerns, including deprivation of enrichment, limited movement, and social stress [9,10].

Although some fur is still sourced from wild-caught animals, approximately 95% comes from animals bred, confined, and killed on commercial fur farms [6,8]. In the U.S., fur farming has sharply declined, with mink farms, for example, decreasing from 236 in 2017 to approximately 100 in 2022 [4]. Yet, the US remains the fifth largest producer of mink pelts in the world; in 2021, U.S. farms still produced about 1.44 million mink pelts valued at nearly USD 60 million [11]. While domestic farming has declined, most fur garments sold in the United States are imported [12]. The majority of these furskin imports consist of mink, fox, and sable pelts [13]. Luxury imports, including astrakhan (Persian lamb, *Swakara*, produced from Karakul lambs killed within hours or days of birth to preserve the distinctive tight curls of the fleece [14,15]), sable (*Martes zibellina*), and lynx (*Lynx lynx*), continue to be popular imported products. Globally, production of fur products remains significant; China, for example, is the largest producer and consumer of fur, generating an estimated 3.5 million mink and fox pelts in 2023, followed by Poland and Russia [16].

The persistence of fur farming and luxury fur markets raises ethical, environmental, and public health concerns. Unlike animal use for food or medicine, fur is produced almost entirely for aesthetic purposes, yet it entails lifelong confinement or, in the case of Karakul lambs, the killing of newborn animals. These moral contradictions explain why advocates, veterinarians, and scientists continue to view the fur trade as an issue of animal welfare, social justice, and planetary health.

### 1.1. Animal Welfare Concerns

Commercial fur farming raises serious welfare concerns including deprivation of enrichment, lifelong confinement in small barren cages, social isolation, and killing methods such as anal, oral, or genital electrocution employed to preserve pelt quality [10]. The European Food Safety Authority (EFSA) has concluded that serious suffering is unavoidable in cage systems for mink, foxes, raccoon dogs, and chinchillas, finding that current housing practices are fundamentally incompatible with these species’ physiological and behavioral needs [17,18].

### 1.2. Environmental Harms

Fur production also generates substantial ecological damage including greenhouse gas emissions, nitrogen and phosphorus runoff leading to eutrophication, and biodiversity threats posed by escaped animals that establish themselves as invasive species [10,19]. Wastewater from farms contaminates rivers and lakes, while tanning and dyeing processes rely on toxic chemicals such as formaldehyde and chromium which are harmful to workers, surrounding ecosystems, and downstream human populations [10,20,21,22]. Empirical evidence also indicates that pollutants from fur farms, including organic pollutants and heavy metals, contaminate nearby freshwater ecosystems, posing risks to both wildlife and human health [10,20,21,22].

### 1.3. Public Health Risks

Public health risks are equally concerning. Overcrowding, close confinement, and constant human–animal contact on fur farms create conditions ripe for zoonotic spillover. Warwick et al. (2023) [10] identify at least 18 endemic pathogens associated with fur farms—including *E. coli*, avian influenza, hepatitis E, and Japanese encephalitis—each with documented or potential cross-species transmission. ActAsia (2024) similarly highlighted the breadth of infectious disease risks linked to fur production [23]. The COVID-19 pandemic underscored the urgency of these concerns: SARS-CoV-2 outbreaks were recorded on mink farms in Denmark, the Netherlands, France, Spain, and the United States, with confirmed mink-to-human transmission [24,25,26]. Multiple studies have warned that fur farms, due to their dense populations of susceptible animals, could serve as viral reservoirs and breeding grounds for future pandemics [24,27].

### 1.4. Political and Policy Responses

The COVID-19 pandemic created new political opportunities for reform [28]. As outbreaks spread, governments introduced emergency measures, including mass culling and permanent closures. Denmark’s 2020 decision to cull its entire mink population epitomized the dramatic shifts in public discourse and policymaking [28]. For several countries, including France, Latvia, and Sweden, COVID-19 helped legislators and advocates reframe fur farming from just an animal welfare issue into a broader public health crisis.

Policy responses have also emerged through international trade law. The World Trade Organization’s ruling in the EC–Seal Products dispute affirmed that trade restrictions based on animal welfare can be justified under the “public morals” exception [29,30]. This decision, widely cited in fur-ban debates, underscores that ethical concerns can override free trade norms. McCulloch (2019) noted that Brexit offered the UK an opportunity to tighten animal welfare laws, with fur imports highlighted as an inconsistency between domestic bans and international trade [31]. In the U.S., California became the first state to enact a comprehensive ban on the sale and manufacture of new fur products in October 2019, with exemptions for used/vintage fur and fur products used for religious purposes [32]. In 2025, Ohio legislators introduced new bills following an investigation into a fur farm charged with animal cruelty, while Massachusetts lawmakers are currently considering two fur-ban bills (SD.712 and HD.2107) [33,34].

Despite widespread public support for fur restrictions, legislative progress within the United States has been limited. California remains the only state to have enacted a comprehensive fur-sales ban (2019), and no comparable state-level prohibitions have since passed [32]. While new bills have been introduced in states such as Massachusetts and Ohio, most proposals have stalled before reaching a floor vote. This stagnation suggests that social and political barriers—including industry lobbying, enforcement complexity, and competing policy priorities—continue to impede reform. Moreover, recent cultural shifts complicate assumptions that fur is universally stigmatized. Recent coverage in The Wall Street Journal [35] and The New York Times [36,37] document a modest resurgence of interest in fur, particularly among younger consumers drawn to vintage and secondhand markets and influenced by celebrity fashion trends. These developments underscore that while opposition to new fur production remains strong, evolving fashion norms may temper policy momentum and challenge traditional stigma-based advocacy approaches.

### 1.5. Consumer Attitudes and Messaging

The changes in policies parallel shifts in consumer attitudes. Research suggests that many consumers, especially younger, pro-environmental millennials, view fur as inconsistent with sustainability values, negatively impacting brand attitudes and purchase intentions [38]. Perceived social stigma, driven by decades of activism against fur, has also transformed fur from a symbol of prestige into a morally tainted product among much of the public, weakening the demand for fur [1]. As ethical interest in animal welfare has increased, faux fur has become an increasingly viable alternative [39]. Yet, the perception and adoption of faux fur is nuanced. While often positioned as a cruelty-free substitute, some research suggests that faux fur may not fully displace demand for real fur. Some consumers, particularly those motivated by fashion status or the desire for authenticity, continue to value real fur’s symbolic prestige [1,39]. Additionally, concerns have been raised regarding the environmental impact of faux fur, as many products have traditionally been petroleum-based and contribute to microplastic pollution [40]. Yet, substantial innovations have been made and newer faux-fur materials increasingly incorporate recycled polyester, plant-based polymers, and bio-derived fibers such as hemp and corn-byproduct blends, resulting in a reduction in greenhouse gas emissions and microplastic shedding [40,41,42].

While much of the outreach to the public and policymakers around fur and fur farming has applied messaging focused on animal welfare implications, increasingly, environmental and public health frames are being utilized. Other persuasive efforts in the United States highlight the sustainability and functionality of faux-fur materials or note that most fur sold domestically originates from largely unregulated international markets in China, Poland, and Russia. Message framing, or presenting an issue in different ways to alter preferences and choices, has been shown to influence and shape public attitudes and behaviors related to animal protection and environmental issues [43,44]. Some studies have found that health- and environmentally framed appeals can be effective in changing consumer behaviors [45]. Other studies exploring animal welfare messages have found that highlighting cruelty significantly reduces willingness to consume animal products compared to environmental or incremental health frames [46,47].

Communication research underscores that tailoring messages to align with audience values, correcting misperceptions of prevailing social norms, and ensuring source credibility are critical to effective persuasion [48]. Within fashion, research has shown that stigma- and norm-based messaging exerts particular influence: when consumers perceive widespread disapproval of fur, intentions to purchase decline sharply (Shin & Jin, 2021 [1]).

While existing survey evidence points to broad baseline support for fur restrictions in both the U.S. and UK [6,7], less is known about public views on policies directly targeting commercial fur farms; the role of beliefs, knowledge, and social norms in shaping opinions; and the relative effectiveness of different messaging strategies. This study addresses these gaps by examining public knowledge, attitudes, perceived norms, and policy support across four northeastern U.S. states—New York, Massachusetts, Connecticut, and New Jersey. These northeastern states were selected because they are active in both fur commerce and policy reform. New York City remains the historic center of the U.S. fur garment industry and continues to host the largest concentration of fur retailers and importers in the country [13,49]. New Jersey also has a longstanding commercial presence in fur manufacturing and trade, particularly through wholesale and distribution channels. Massachusetts and Connecticut were included because each has recently introduced or debated legislation to prohibit the sale of new fur products: Massachusetts SD.712/HD.2107 (2025 session) and prior Connecticut proposals such as Raised Bill No. 5330 (2024 session), although neither has yet been enacted [50,51]. These states represent a strategically relevant cross-section of markets where fur commerce remains visible and policy reform efforts are ongoing, allowing for analysis of how economic, political, and attitudinal contexts interact in shaping support for fur bans. State-level analysis is particularly relevant because animal welfare legislation in the United States is primarily enacted at the state level, and understanding public attitudes within these jurisdictions can inform policymakers and advocacy organizations currently debating fur-related bills. Building on evidence that animal welfare, environmental, public health, stigma, and faux fur alternative frames may influence attitudes in distinct ways, our study was designed to test which messages are most persuasive. We also explored how demographic and social–psychological characteristics moderate responsiveness.

Specifically, we addressed four research questions:

1. What are public knowledge, attitudes, perceived social norms, and beliefs toward fur products, faux fur, and fur farming in four northeastern states?
2. To what extent does the public support state-level policies banning the sale of fur overall and from commercial fur farms specifically, and how does this support vary by state?
3. To what extent is support for state-level bans influenced by exposure to different types of messaging (animal welfare detailing current farming harms, environmental harm, public health risk, economic arguments, faux fur alternative framing, or social norms/stigma), and do these effects differ by political affiliation, age, or identity orientation?
4. What are the most important social–psychological (beliefs, attitudes, perceived norms, stigma, knowledge of harms) and demographic predictors of support for state-level fur bans?

## 2. Materials and Methods

### 2.1. Sample Recruitment

Consistent with prior surveys of public opinion on fur [7], we recruited a demographically representative sample of U.S. adults from an online panel provider. Our target sample size was 2000 respondents, distributed evenly across four states (Connecticut, Massachusetts, New Jersey, and New York) to obtain approximately 500 participants per state and at least 285 respondents per message condition (six messages plus a no-message control).

We partnered with Cint (formerly Lucid) (Stockholm, Sweden) as the panel provider based on the analysis and subsequent recommendation by Stagnaro et al. (2024) [52] for researchers studying social and political content with shorter treatments and simpler measures. In line with best practices for ensuring data quality in online survey research, we implemented two front-end attention checks to identify inattentive or automated responses. These were simple instructed-response items (e.g., “Please select ‘slightly agree’ for this statement”) designed to verify that participants were reading questions carefully. Attention checks are widely recommended for improving the validity of online data collected through panels such as Cint and Prolific, where inattentive or automated responses can otherwise compromise reliability [53,54,55]. Respondents who failed one or both attention checks (*n* = 2840) or produced nonsensical or bot-like open-text answers (*n* = 42) were excluded from analysis, resulting in a final analytic sample of 2014 respondents: 506 from Connecticut, 504 from Massachusetts, 500 from New Jersey, and 504 from New York. Although this exclusion rate was higher than typical for smaller-scale surveys, quality screening is consistent with recent large online studies in which 40–60% of raw responses fail attention checks [56]. Importantly, the demographic characteristics of the retained sample closely matched state and national benchmarks (Table 1), suggesting that exclusions did not meaningfully bias representativeness.

### 2.2. Survey

The anonymous survey was developed in consultation with U.S.-based experts in outreach, policy, and education related to fur and fur farming. The instrument was pilot-tested to evaluate wording, timing, and survey logic with feedback informing subsequent revisions. To reduce bias and improve data quality, we randomized item order and included attention checks. All participants provided informed consent, and ethics approval was received from the Institutional Review Board of Colorado State University (#6701, 3/3/2025).

### 2.3. Behaviors and Attitudes Toward Fur and Faux Fur

Respondents were asked about their likelihood of purchasing new clothing containing fur from animals raised on fur farms and used/vintage fur clothing. They were also asked how acceptable they consider new and used/vintage fur clothing, and whether they would be more or less likely to shop at a store that voluntarily stopped selling fur products. Participants reported whether they purchase faux/fake fur clothing, whether they have sought suitable alternatives to real fur, and how easy or difficult they found it to obtain such alternatives (i.e., behavioral control).

### 2.4. Social Norms

Following Niemiec et al. [58], we examined descriptive and injunctive social norms related to purchasing fur. Following the suggestions of Rimal and Lapinski [59], we examined norms for reference groups that varied in social distance. To assess perceived descriptive social norms among their most proximal social groups, participants were asked to indicate how likely their friends and family would be to purchase fur clothing. To assess injunctive social norms, respondents reported how they believed their friends would feel if they themselves purchased fur. Broader perceived social norms were captured by asking respondents to estimate the percentage of U.S. residents who purchase fur products and the percentage who disapprove of fur use in clothing and accessories.

### 2.5. Knowledge

To measure objective knowledge of fur farming, respondents were asked

1. To list two common animal species farmed for fur in the United States (open text).
2. Whether most fur originates from farmed animals or from wild-trapped animals (farmed animals, wild-trapped animals, unsure).
3. Whether they had seen, read, or heard about campaigns to stop the sale of fur products (yes, no, unsure).

### 2.6. Beliefs

Respondents indicated their agreement level using a 5-point Likert scale (1 = strongly disagree, 5 = strongly agree), with 12 belief statements addressing fur, faux fur, and the impacts of fur farming on animal welfare, the environment, and public health. Examples include “To me, wearing fur is a symbol of wealth and social status” and “Buying fur is a right that all people should have”.

### 2.7. Message Conditions

Participants were randomly assigned to one of six informational message conditions or a no-message control. Messages were designed to reflect common advocacy frames and were reviewed by experts to ensure accuracy and relevance. Each message was preceded by the statement “Before we ask you more questions, we’d like to share a bit more information with you about the fur farm industry and hear what you think of this information.”

The messages included the following:

- Animal welfare: Described 16 categories of welfare concerns prevalent on fur farms from Warwick et al. (2023) [10], including barren housing, stress, abnormal behaviors, disease, and inhumane killing methods (e.g., electrocution).
  ○ “A recent scientific study found that despite numerous efforts to systematically monitor and control animal welfare at fur farms, practices continue failing to meet animal welfare standards. This study found 16 categories of animal welfare concern (e.g., animals being denied access to things they need or want, stress, abnormal behaviors, unsanitary conditions, forced obesity, and high levels of sickness, disease, and death) are prevalent in fur farms. Animals are deprived of enrichment, forced to stand and rest on floors of bare wire cages, spend their entire lives in small, barren, wire-mesh cages too small for normal movement, and are deprived of contact with individuals of the same species. Further, in order to protect their fur, animals raised for fur are often killed by oral, anal, or genital electrocution.”
- Environmental: Highlighted four categories of environmental concern described in Warwick et al. (2023) [10]: greenhouse gas emissions, invasive species (15–38% originating from fur farming), water pollution from nitrogen and phosphorus runoff, and toxic chemicals (e.g., formaldehyde, chromium) used in tanning and dyeing.
  ○ “A recent scientific study identified four main categories of environmental concern—greenhouse gas emissions, invasive species, toxic chemicals, and water quality impacts—associated with fur farming. An invasive species is a non-native mammal that reproduces quickly and causes harm to the environment, economy, and/or human health. It is estimated that between 15% and 38% of all invasive mammal species originate from fur farming. Additionally, there is a higher concentration of nitrogen (N) and phosphorus (P) in the manure of mink compared to certain livestock. When these N and P salts are washed into water courses, aquatic plants and algae overgrow, which leads to depletion of oxygen and degradation of the ecosystem. Furthermore, fur is often tanned and dyed using toxic chemicals like formaldehyde and chromium, which can pose significant environmental and health risks, including pollution of soil and water and potential harm to workers and consumers.”
- Public health: Outlined the risk of zoonotic disease spread due to confinement and stress, citing influenza, hepatitis E, Japanese encephalitis, and at least 18 pathogens associated with fur farms (described in Warwick et al. (2023) [10]).
  ○ “Fur farming can negatively impact public health by allowing pathogens-viruses, bacteria, or fungi that can cause disease-to spread from animals to humans. Because animals are confined in small spaces and are stressed by confinement, it makes it easier for viruses to spread. Animals on fur farms have been found to carry viruses that cause influenza as well as hepatitis E and Japanese encephalitis. A recent scientific review paper identified 18 reported endemic pathogens and diseases (e.g., Botulism, E. coli infection, avian flu) with confirmed or potential zoonotic and cross-species implications in fur farms.”
- Faux fur alternatives: Presented examples of innovative and sustainable (i.e., plant-based and recycled) faux fur alternatives (e.g., Sorona, hemp-based fibers, recycled polyester, Koba bio-based blends) increasingly used by luxury fashion brands.
  ○ “Clothing that provides warmth and insulation that is comparable to fur from animals is widely available. Several luxury fashion designers (e.g., Stella McCartney, Gucci, Prada, and Alexander McQueen) are using faux/fake fur alternatives that have the same look and feel as fur. While alternatives to fur used to be petroleum based, faux/fake furs are now environmentally friendly and made from plant-based materials. Examples include “Sorona” (derived from corn), hemp-based faux fur, recycled polyester fibers, and innovative bio-based faux furs like “Koba” which use a blend of plant-based materials and recycled polymers, significantly reducing the environmental impact.”
- Economics: Emphasized that most fur sold in the U.S. originates from unregulated farms abroad, particularly in China and Russia, with China producing 3.5 million mink and fox pelts in 2023.
  ○ “Consumers purchasing fur in the United States are likely not supporting US farms, but rather supporting an unregulated fur industry in countries like China and Russia. China is by far the biggest producer and consumer of fur; in 2023, China had an output of 3.5 million mink and fox pelts, followed by Poland and Russia.”
- Social norms: Shared survey data [7] showing that 73% of likely U.S. voters are concerned about fur use and over 50% support banning fur sales.
  ○ “Because of increased knowledge on the environmental, animal welfare, and public health impacts of fur farms, the majority of the American public is concerned about the use of fur and supports policies to ban fur sales. One US national poll conducted in 2022 found that 73% of likely voters are concerned about the use of animal fur in clothing apparel and accessories like mink coats. Over 50% of likely voters would support a policy banning the sales of fur.”

Each respondent was asked to read their assigned message and provide a brief description of the key takeaway they would share with others. These qualitative responses served as a manipulation and attention check to confirm message comprehension. We reviewed all open-ended responses for coherence and relevance to the assigned message (e.g., references to animal welfare, environmental, or public-health themes). Responses that were blank, nonsensical, or clearly unrelated to the message content were excluded from the analysis. The messages were presented after questions on knowledge, attitudes, social norms, and beliefs, but before outcome questions regarding policy support to assess the message’s effect on support for fur bans.

### 2.8. Support for Fur Ban Policies

Prior to questions pertaining to support for fur ban policies, all respondents were provided with the following background information on proposed state-level bans:

“Some stakeholders are working to pass local and state legislation to stop the sale of new fur products. This legislation would prevent the sale of clothing and accessories made from fur, defined as animal hair that remains attached to the skin (e.g., fur coats, fur trim on bags/hats, or fur rugs). It would not ban the sale of loose hair items, such as felt hats, paintbrushes, or fishing lures.”

Respondents were then asked to indicate their support for a ban on the sale of fur from any animal (wild or farmed) and a ban on the sale of fur from animals raised on commercial fur farms using a 5-point Likert scale with 1 = very unsupportive and 5 = very supportive:

Those who indicated they were neutral or unsupportive were asked whether they would support a ban if exemptions were included for religious purposes (e.g., mink fur hats), for Native American tribal or cultural uses, or if no exceptions were made. Finally, participants reported whether a candidate’s support for a fur ban would make them less or more likely to vote for that candidate, or if there would be no difference.

### 2.9. Analysis

To answer research questions #1 and #2, we conducted basic descriptive analyses of behaviors, beliefs, attitudes, social norms, and policy support for our overall sample and, for many variables, by state. Given the slight differences between our sample demographics and actual demographics for the four states surveyed, we conducted descriptive analyses unweighted and weighted for state-level demographics (Table 1). To answer research questions #3 and #4, we used targeted maximum likelihood estimation [60] to model the impact of message condition, behaviors, beliefs, attitudes, social norms, and demographics on policy support (i.e., both support of a ban on all fur and a ban on fur coming from animals raised in commercial fur farms).

We estimated the effect of the messaging interventions compared to the control arm and to each other on support for legislation banning sale of all fur and fur from commercial farms (research question #3) both unadjusted and adjusted for covariates. We conducted adjusted analyses using targeted maximum likelihood estimation which allows for flexible covariate adjustment (i.e., allowing for interactions and non-linearity) with machine learning models. We included the following covariates in the models: all demographic variables as well as all the belief items related to fur and faux fur, behaviors (i.e., whether or not they have purchased fur or faux fur and likelihood of purchasing new or used fur), perceived acceptability of fur, and all the social norms questions. We also ran adjusted linear regression models as a sensitivity analysis and to examine the relative importance of different adjustment covariates on support for legislation (research question #4).

## 3. Results

### 3.1. Description of the Sample

Our final sample size was 2014: 506 from Connecticut, 504 from Massachusetts, 500 from New Jersey, and 504 from New York. Our sample was generally representative of United States demographics (Table 1); although we had slightly fewer republicans, higher education, and lower income than estimates of the overall US population. When compared to the average demographics of the four states, our sample had similar education, lower average income, and more republicans. Overall, participants were 53% female and 46% male. Eighteen percent were between 25 and 34 years of age, and twenty-one percent were 65 years or older. Forty-four percent of respondents held a bachelor’s degree or higher, and 28% reported annual household incomes above USD 100,000. The racial and ethnic composition of the sample was primarily White/Caucasian (74%) and Black/African American (16%) (Table 1). We therefore reported our outcome variables unweighted and weighted by demographics to be representative of the four states’ demographics. To conduct the weighting, we used iterative proportional fitting (ranking) based on political party membership and average demographics across the four states [61]. In addition to age, race, education, and political affiliation, we asked participants about pet ownership. We found that 64.5% of the sample currently owned a pet, 27.6% reported owning a pet in the past but not currently having a pet, and 7.9% reported never owning a pet. These numbers are similar to national data; for example, the American Pet Products Association (APPA) found that 71% of U.S. households owned a pet in 2024–2025 [62].

### 3.2. Knowledge, Beliefs, and Behaviors Related to Fur, Fur Farming, and Faux Fur

Over half of the sample (55.9%) did not own any fur products. Respondents from Connecticut were least likely to own fur products (61.7% did not own fur products) while respondents from New Jersey were more likely to own fur products (51.0% did not own fur products). Approximately 29.4% of respondents indicated that they would be somewhat or very likely to purchase new clothing containing fur from animals raised on fur farms, while 31.5% indicated they would be somewhat or very likely to purchase used/vintage clothing containing fur from animals raised on fur farms. There were only slight variations in likelihood of purchasing new or used/vintage fur by state (Table 2).

### 3.3. Perceptions of Acceptability

Approximately 48.3% of participants believed that purchasing new clothing containing fur was somewhat/very unacceptable, and 30.8% believed it was somewhat/very acceptable (20.9% were neutral). A total of 37.5% believed that purchasing used/vintage clothing containing fur was somewhat/very unacceptable, while 38.7% believed it was somewhat/very acceptable (23.8% were neutral). When asked about a store that voluntarily agrees to stop selling clothing and other products that contain fur from animals, approximately 42.1% said they would be more likely to shop there, 10.0% said less likely, and 47.9% said it would make no difference.

### 3.4. Social Perceptions and Normative Beliefs

Approximately 28.7% of participants reported believing that their friends and 31.7% believing that their family would be somewhat or very likely to purchase new clothing or used/vintage clothing containing fur from animals raised on fur farms. When asked how they thought their friends would feel if they were to purchase new clothing containing fur from animals, 23.1% felt their friends would respond somewhat or very positively, and 39.2% felt their friends would respond somewhat or very negatively (37.7% were neutral). On average, respondents reported believing that 41.5% of US residents purchase new clothing/accessories containing fur from animals. In addition, 56.6% of respondents reported believing that Americans disapprove of the use of animal fur in clothing/accessories.

### 3.5. Faux Fur Attitudes and Use

Regarding faux fur, approximately 10.4% of respondents reported that they often purchase clothing with faux/fake fur, 45.2% said occasionally, 38.7% said never, and 5.7% said they were unsure. When asked whether they had tried to find clothing or other products that are suitable alternatives to fur, 13.9% said often, 37.1% said sometimes, 44.6% said never, and 4.4% were unsure. Those who had tried to find fur alternatives were asked how easy or difficult it is to find clothing or other products that are suitable alternatives to fur. A total of 14.6% responded very easy, 24.3% responded somewhat easy, 8.0% responded somewhat difficult, 1.1% responded very difficult, and 7.6% responded unsure (Table 3).

Across all four states, the majority of respondents (62.2%) reported believing that there are suitable faux/fake fur alternatives. Less than a third of respondents (26.3%) believed that faux/fake fur alternatives are bad for the environment, and approximately one third (31.8%) believed that faux/fake fur alternatives are not as fashionable or desirable as fur (Table 3).

When respondents were asked to share 1–2 sentences about their perspective on faux/fake fur (including why they are or are not interested in purchasing items with faux/fake fur), common answers included the following:

- Don’t buy fake things;
- Fake fur is tacky/cheap;
- Faux fur is a good alternative;
- Don’t wear fur in general;
- Like authentic fur for longevity;
- Like how faux fur doesn’t involve killing an animal;
- Faux fur is itchy;
- Faux fur nowhere near quality of real fur;
- Faux fur less expensive.

### 3.6. Knowledge of Fur Farming and Animal Species Used

When respondents were asked to list the two most common species/types of animals that are farmed for fur in the United States, 32.2% included mink in their answers and 33.3% included fox. Only 17.0% mentioned both mink and fox (the correct answers). Other common responses were bear, chinchilla, sheep, lion, tiger, and wolf.

When respondents were asked whether most fur used in clothing and other products come from animals farmed specifically for their fur or from animals that are trapped in the wild, 44.0% selected animals farmed specifically for their fur (the correct answer), 8.8% selected animals trapped in the wild, and 47.2% selected unsure.

Approximately 28.3% of participants indicated that they had seen, read, or heard about efforts to stop the sale of clothing and other products containing fur from animals, while 62.2% indicated no, and 9.5% indicated they were unsure.

### 3.7. Beliefs About Fur and Fur Farming

Respondents were asked to indicate their agreement with a variety of belief statements in support of or in opposition to fur and fur farming (Table 3). Approximately 41.5% of respondents believed that wearing fur is a symbol of wealth and social status and 42.0% felt that buying fur is a right that all people should have. Approximately 43.3% believed that the government shouldn’t regulate whether people can purchase fur products.

The majority (60.7%) believed that fur farming involves cruel and inhumane treatment of animals. However, only 41.3% of respondents agreed that fur farming is bad for the environment and 35.6% felt that it can lead to disease that can spread to humans. Approximately 39.8% believed that fur farms in the US are regulated to ensure basic animal welfare standards are met (Table 3).

### 3.8. Overall Support for State Policies Banning Fur

#### 3.8.1. Control Group (No Messaging)

When we examined support among those in our control group (*n* = 285) who did not receive any of the informational messages about fur without weighting our sample, 56.1% were somewhat or very supportive of a state ban on the sale of fur from any animal (caught in the wild or from commercial fur farms), and 58.6% were somewhat or very supportive of a state ban on the sale of fur from animals raised on commercial fur farms. When we weighted our sample to reflect the average demographics of the four states, 59.1% were somewhat or very supportive of a state ban on the sale of fur from any animal, and 61.3% were somewhat or very supportive of a state ban on the sale of fur from animals raised on commercial fur farms.

#### 3.8.2. Full Sample

Across our full sample (including individuals who received various messages about the impacts of fur farming and faux fur alternatives; *n* = 2014), without weighting, 61.6% indicated that they would be somewhat or very supportive of a state ban on the sale of fur from any animal (caught in the wild or from commercial fur farms). With weighting to reflect average demographics across the states, 64.7% were somewhat or very supportive of such a ban. Comparing across the four states, support was greatest in Massachusetts: 62.3% (unweighted) and 66.1% (weighted for the state’s demographics) of respondents were somewhat or very supportive, followed by Connecticut (61.9%, unweighted; 64.8% weighted), New York (61.5% unweighted; 64.5% weighted), and New Jersey (60.8% unweighted; 64.4% weighted). Across our full sample, without weighting, 61.8% of participants indicated that they would be somewhat or very supportive of legislation to stop the sale of new clothing and other products containing fur from animals raised in commercial fur farms (i.e., with animals held in captivity). With weighting to reflect average demographics across the states, 64.9% were somewhat or very supportive. Respondents from all four states had similar levels of support: Massachusetts and New Jersey had slightly more support (both 62.4% somewhat or very supportive, unweighted; Massachusetts 66.2% weighted and New Jersey 64.5% weighted) followed by New York (61.5% unweighted; 64.4% weighted), and Connecticut (60.9% unweighted; 64.9% weighted). Additionally, 11.7% (unweighted) indicated that they would become somewhat or very supportive of such legislation if there was an exemption of fur products used for religious purposes (e.g., religious hats made out of mink fur) and 16.3% indicated they would become somewhat or very supportive if there was an exemption of fur products used for Native American tribal or cultural purposes.

### 3.9. Impact on Voting Preferences

Among our full sample, when asked about a political candidate who supported a ban on the sale of fur from any animal, 43.1% unweighted (43.9% weighted) said that it would make them more likely to vote for them, 42.9% unweighted (41.4% weighted) said it would make no difference on their vote, and 13.9% unweighted (14.8% weighted) said that the candidates’ support of a ban would make them less likely to vote for them.

This support was similar when broken down by state, weighted by state demographics. In Massachusetts, 47.5% reported they would be more likely, 39.1% reported no difference, and 13.2% reported they would be less likely to vote for them. In Connecticut, 46.1% reported they would be more likely, 40.2% reported no difference, and 13.5% reported they would be less likely to vote for them. In New York, 45.8% reported they would be more likely, 40.4% reported no difference, and 13.6% reported they would be less likely to vote for them. In New Jersey, 45.6% reported they would be more likely, 40.2% reported no difference, and 14.0% reported they would be less likely to vote for them.

### 3.10. Impact of Messaging

**Figure 1:**
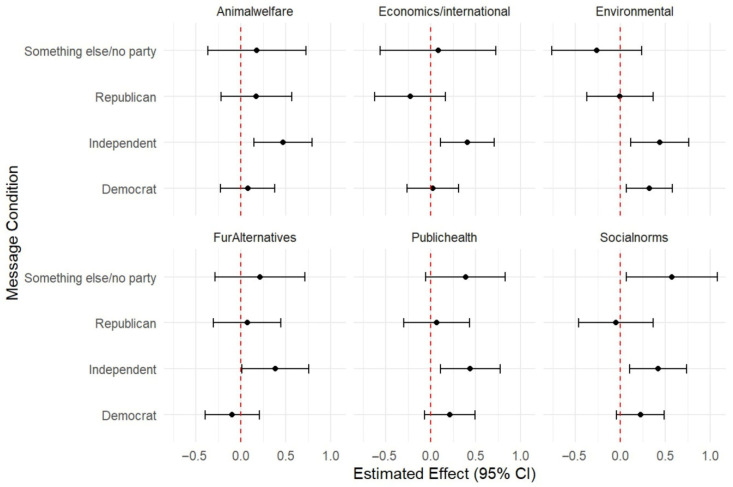
TMLE estimates of the impact of message condition on support for legislation banning fur from all animals for sub-groups of political affiliation.

**Figure 2:**
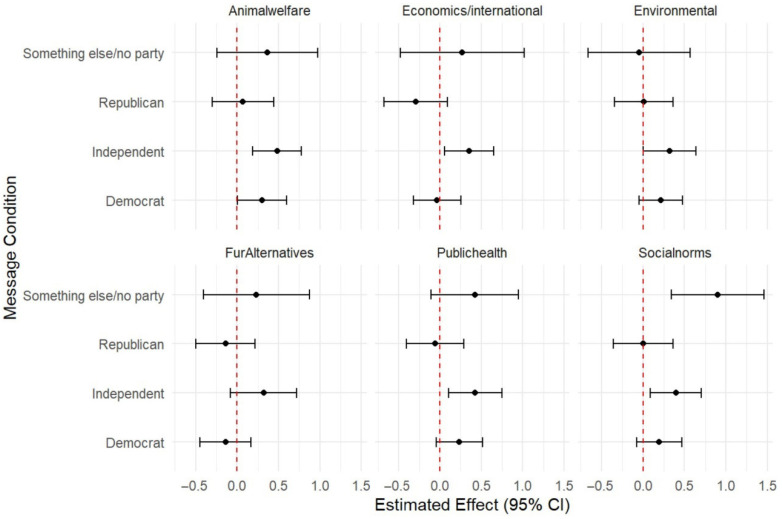
TMLE estimates of the impact of message condition on support for legislation banning fur from commercial farms for sub-groups of political affiliation.

The social norms, animal welfare, public health, and environmental impact messages all had a significant positive effect on support for legislation banning fur from an animal caught in the wild or from a commercial fur farm (Table 4) and legislation banning fur from animals raised in commercial fur farms (Table 5) compared to a no-message control when adjusting for social–psychological and demographic covariates in the TMLE machine learning models as well as linear regression (Figure 1 and Figure 2).

Contrasting the messages with one another revealed that the social norms, public health, environmental, and animal welfare messages were significantly more effective at increasing support for bans of fur (overall and for fur from commercial farms) compared to the economics message. In addition, for predicting support of bans of fur from commercial farms, the animal welfare, environmental, social norms, and public health messages were significantly more effective than the faux fur alternatives message. For predicting support of bans of fur overall, the environmental message was significantly more effective than the faux fur alternatives message.

In a subgroup analysis of message condition by political affiliation, all four significant message conditions for the full sample (i.e., social norms, animal welfare, public health and environmental impacts) were significant for the sub-sample of respondents who identified as politically independent (Figure 1 and Figure 2).

The economics message also had a significant positive impact on support for legislation for the subgroup of independents, and the social norms message had a significant positive impact on those who did not identify as political or with a party. Only the environmental message had a significant impact on democrats (for the outcome of banning fur from all animals; Figure 1), while none of the messages had a significant impact on republicans (Figure 1 and Figure 2).

### 3.11. Predictors of Support

**Figure 3:**
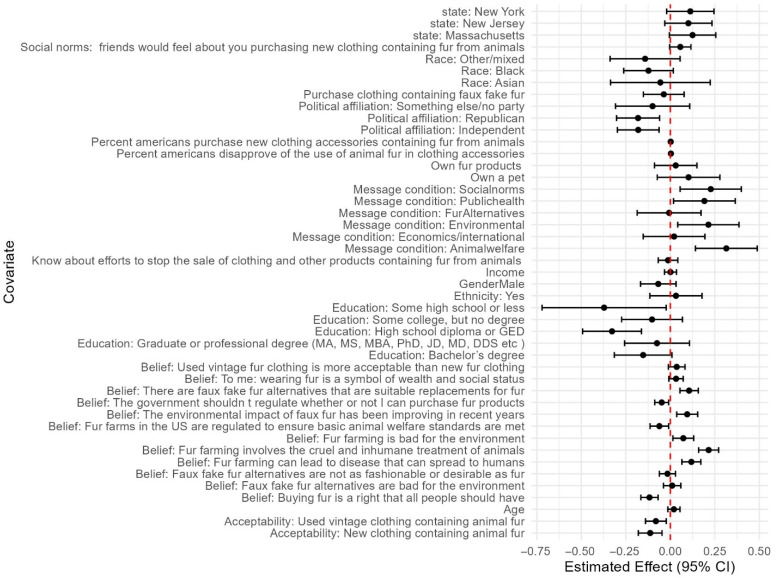
Regression coefficients and 95% confidence intervals from all messages. Conditions, demographics, and social–psychological variables from multiple linear regression predicting support for legislation banning fur from all animals.

**Figure 4:**
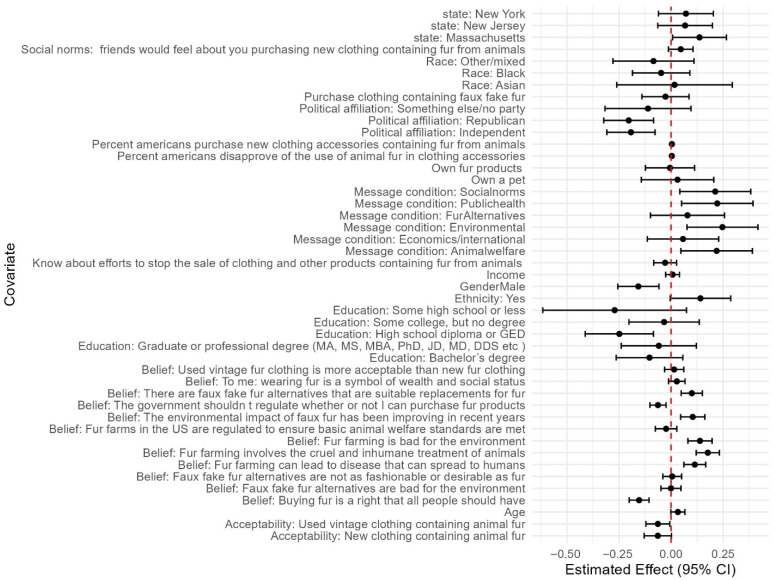
Regression coefficients and 95% confidence intervals from all message conditions, demographics, and social–psychological variables from multiple linear regression predicting support for legislation banning fur from commercial fur farms.

Our linear regressions identified numerous social–psychological beliefs and demographics, in addition to message conditions, that impacted support for legislation banning fur from any animals and from fur farms (Figure 3 and Figure 4). In addition to the significant positive effect of the message conditions discussed above (social norms, public health, environmental, and animal welfare impacts), the following variables were significant positive predictors of both support for banning fur from all animals and from commercial fur farms (Figure 3 and Figure 4): beliefs that fur farming involves the cruel and inhumane treatment of animals; that fur farming is bad for the environment; that fur farming can lead to disease that can spread to humans; that there are faux fur alternatives that are suitable replacements for fur; that the environmental impacts of faux fur have been improving in recent years; and the perceived percentage of Americans that disapprove of the use of animal fur in clothing.

The following were significant negative predictors of both support for legislation banning fur from all animals and from commercial fur farms: the belief that buying fur is a right that all people should have; identifying as republican; identifying as politically independent; the belief that government should not regulate whether or not someone can buy fur products; having a high school diploma as the highest level of education; and believing that buying used clothing containing fur was more acceptable.

Being from Massachusetts and the perceived percentage of Americans that purchase new clothing/accessories contacting fur was a significant positive predictor only of support for a ban on fur from all animals. Identifying as male was a significant negative predictor only of support for a ban on fur from all animals.

Greater perceived acceptability of buying new clothing containing fur and beliefs that fur farms in the US are regulated to ensure basic animal welfare standards are met were negatively associated only with support for a ban of fur from animals raised on commercial farms.

## 4. Discussion

The fact that more than an ever-increasing number of countries, as well as US states and cities, have implemented fur bans [4,19] illuminate the fact that perceptions about fur are changing. The COVID-19 pandemic accelerated the process by exposing fur farms as significant zoonotic risk environments [63,64,65]. More than ever, fur farming is being viewed, not only as an animal welfare issue, but a public health hazard [63]. Within this evolving environment, our survey and message-experiment results offer several insights into how residents in northeastern U.S. states think about fur, their support for policies to ban the sale of fur, and the impact of message framing on policy support.

### 4.1. Public Attitudes Toward Fur and Faux Fur

Our data support the premise that social stigma and ethical consumerism have changed the perception of fur. Although historically fur has signified luxury and status [1,38,39], the majority of our respondents indicated they do not own fur products, and fewer than one in three expressed likelihood of purchasing new or vintage fur in the future.

In addition, nearly half of our participants reported feeling new-fur purchases are unacceptable, and 39% said they would expect negative reactions from their friends and family if they purchased clothing containing fur. Despite their own views about fur, as well as their friends’ and families’, respondents tended to overestimate how many Americans buy fur. Correcting this pluralistic ignorance (a misperception in which a minority position on a given topic is wrongly perceived to be the majority position [66,67]) could help advance support for fur bans. When people overestimate how many others buy or support fur, they may remain silent about their own opposition, thereby reinforcing this false notion. By educating the public that most Americans do not purchase fur and that fur farming faces broad disapproval, advocates can embolden individuals to voice their true views. Research shows that norm-correction interventions can effectively counteract pluralistic ignorance and shift both attitudes and behaviors [68,69]. Indeed, our social norms messaging, which highlighted how the majority of the public supports bans on fur sales, did have a positive and significant effect on support, suggesting that normative messaging may be particularly effective on this issue.

When asked about faux fur, responses were more ambivalent. While a majority believed faux alternatives could substitute for real fur, a sizable minority raised doubts around their environmental footprint, fashionability, or authenticity (i.e., does not look/feel like real fur). Additionally, the faux fur messaging that we tested, which focused on the sustainability of faux fur alternatives, did not significantly impact support for fur bans. These results mirror literature showing that consumers wrestle with faux fur trade-offs including ethics, aesthetics, and product quality [1,39]. Qualitative responses underscored these divides: while some respondents dismissed faux fur as “cheap” or “inauthentic,” others valued it as a cruelty-free option. Addressing this ambivalence may require leveraging fashion industry leadership to normalize faux fur as stylish, aspirational, and sustainable. Research suggests that consumer acceptance of sustainable fashion alternatives can be strengthened through credible information, labeling, and positive identity signaling [70,71].

### 4.2. Knowledge Gaps and Misconceptions About Fur Farming

Despite heightened media attention, knowledge gaps about the process of fur production remain. Less than half of respondents correctly understood that most fur comes from farmed animals and only about one-third named mink or fox as primary species. In addition, nearly 40% of participants believed U.S. fur farms are regulated to ensure welfare standards. In reality, there are no federal laws that specifically govern the welfare of animals on fur farms, and regulations in most states are weak or nonexistent [10,11,72]. Correcting these misperceptions about regulation is critical, as consumers often rely on presumed regulation when evaluating the legitimacy of controversial industries [73,74]. Additionally, we found that the belief that fur farms are regulated was negatively associated with support for a ban of fur from commercial farms; suggesting that correcting this belief is critical for gaining policy support.

While cruelty was broadly recognized (i.e., 61% agreed fur farms involve inhumane treatment), fewer respondents associated fur farming with environmental damage or zoonotic risks. Efforts to educate people about the mounting scientific evidence linking fur operations to ecosystem pollution, chemical runoff, and pathogen emergence [10,20,22] is needed. Public education on these detrimental effects could lead to more robust support for regulatory interventions.

### 4.3. Legislative Support in Practice

Even without messaging, support for state-level bans was broad: ~56–61% of control-group respondents favored banning fur sales (overall and from commercial farms). Weighted estimates (adjusted for state demographics) rose slightly to ~59–65%. This aligns with national polling showing a majority of people support fur restrictions [6,7].

State-level differences were modest, with Massachusetts often showing the highest levels of support. Exemptions for religious or tribal use boosted backing among some respondents, but the majority indicated support with a full ban. In addition, more than 40% of respondents said they would be more likely to vote for a candidate backing a fur ban and only 14% said they would be less likely. Overall, these results suggest a strong social landscape backing policy action on fur in the Northeastern United States.

### 4.4. The Power of Framing: Messaging Effects and Subgroup Variation

Our study found that animal welfare, environmental, public health, and social norms messaging significantly increased policy support, whereas economics and faux fur alternatives messages did not. These patterns resonate with comparative framing literature in food, climate, and animal advocacy, where moral and risk frames tend to outperform economic persuasion [45,46,47]. Our social norms message, in particular, underscores how highlighting collective disapproval or shifting majority attitudes can tip individual support [48].

Our subgroup results add nuance to these results. Among political independents, all messages, including the economics frame, boosted support, suggesting these voters are more malleable. Among democrats, only the environmental frame held sway; among republicans, none of the messages had an impact. These results echo broader challenges in bridging party divides on environmental and welfare issues [47,75] and suggest that commonly held values (e.g., shared public health risk) may be needed to reach those less supportive of fur bans.

### 4.5. Predictors of Policy Support

Beyond messaging, regression models highlighted several beliefs that impact perceptions of fur bans. Positive predictors included conviction that fur farming is cruel, environmentally harmful, and disease-prone, and that faux alternatives are viable. These results suggest that the most fruitful targets for persuasion may be beliefs about cruelty, ecological damage, and zoonotic risk—especially when paired with norms or identity cues—which corresponds with the results of our messaging experiment. Courts, advocates, and legislators may gain more traction by emphasizing harm and risk rather than cost or choice arguments.

### 4.6. Policy and Advocacy Implications

The findings from this study offer several important directions for policy and advocacy. First, the strong baseline support across states suggests fertile ground for legislative initiatives, indicating that policymakers may be responding to an electorate already inclined toward fur reform. To build on this momentum, messaging strategies should emphasize value-driven frames—particularly those focused on animal welfare, environmental sustainability, and public health risks—rather than economic arguments, which proved less persuasive. At the same time, correcting common misconceptions is essential, especially the widespread but inaccurate belief that U.S. fur farms are adequately regulated. Educational campaigns highlighting that these farms are largely unregulated, environmentally harmful, and a zoonotic risk is needed to increase public understanding of the fur industry.

Leveraging perceptions of social norms is also critical: highlighting the widespread disapproval of fur could amplify stigma effects and accelerate the cultural shift away from fur use. Targeted outreach may be particularly effective with political independents, who demonstrated greater responsiveness to messaging. While none of our messages had a significant impact on a republican audience, moral foundations theory suggests that purity-focused framing may be most impactful for republican values [76]. Purity-focused framing demonstrating how fur farms act as both viral reservoirs and polluting facilities [63,64] could therefore potentially help move them towards supporting fur bans.

Despite this broad base of public support, meaningful policy change in the United States has been slow to materialize. California remains the only state to have enacted a comprehensive fur-sales ban (2019), and subsequent legislative proposals in Massachusetts, Connecticut, and other states have yet to advance beyond committee. This policy stagnation likely reflects a combination of political, economic, and cultural factors. Powerful trade associations and fashion industry stakeholders continue to exert lobbying influence at the state level, framing bans as threats to small businesses and consumer freedom. Socially, recent resurgences of interest in vintage and secondhand fur, amplified by celebrity influence and resale platforms, illustrate that fur’s cultural meaning remains contested. These forces help explain why high levels of public disapproval have not yet translated into widespread policy adoption. Future research should therefore examine the political and economic dynamics that mediate the translation of public attitudes into legislative outcomes, including how advocacy messaging interacts with industry opposition and evolving fashion trends.

### 4.7. Limitations and Opportunities for Future Research

Despite its strengths, several limitations of this study should be acknowledged. First, the survey relied on self-reported attitudes and behaviors, which are vulnerable to social desirability bias [77]. Second, although post-stratification weights were applied to approximate age, gender, education, and political affiliation distributions across the four target states, weighting cannot fully correct for the fact that these states are, on average, wealthier and more Democratic-leaning than the U.S. overall. Consequently, the findings may not generalize to regions with different socioeconomic or political profiles. Third, all four states are more urbanized than the national average, and the survey did not capture participants’ specific geographic residence (e.g., urban, suburban, or rural), which may influence attitudes toward wildlife use and policy. Fourth, the survey did not assess whether respondents or their household members hunted or trapped animals, a factor that may shape beliefs about the fur trade and related legislation. Fifth, although the online panel provider (Cint) allowed for rapid and diverse recruitment, online surveys have inherent constraints: respondents may be more digitally literate, frequent survey-takers, or more politically engaged than the general population, introducing potential selection bias [78]. Sixth, message exposure was limited to a single, brief encounter, and the messages were standardized across topics rather than varying in tone or intensity. Real-world advocacy campaigns often differ substantially in emotional valence, ranging from educational or informational appeals to graphic or confrontational imagery, which may influence engagement and persuasive impact. Finally, because the survey design was cross-sectional and short-term, we cannot assess the durability of opinion change or whether shifts in support translate into real-world actions such as voting, advocacy, or consumer behavior [79]. Future work should track how opinions evolve over time (e.g., pre- and post-ban proposals), incorporate measures of rurality and hunting participation, and experimentally compare advocacy frames that differ in emotional intensity or style. Comparative studies across countries or states with existing fur bans may also help identify which approaches most effectively drive policy change [6,63].

## 5. Conclusions

In sum, public sentiment in the U.S. Northeast shows strong support for banning fur sales, and carefully designed messaging can bolster that backing—especially when rooted in moral, ecological, or health appeals. Beliefs about cruelty, environmental damage, and disease risk can be effective tools for persuasion, and social norms can amplify their impact. As global momentum against the fur industry intensifies, our findings can help advocates, policymakers, and communicators hasten the adoption of fur ban policies.

## Figures and Tables

**Table 1:** Comparison of demographics of the sample with US nationwide demographics (* political party information varies based on polling company; our estimates for the US below are from Ballotpedia data from March 2025 [57]).

	US Nationwide Demographics	Average Demographics Across MA, CT, NJ, and NY	Survey Sample Demographics
% Bachelor’s degree or higher	38%	43%	44%
% Household income USD 100,000 or higher	34%	46%	28%
% 65 years old	17%	18%	21%
% 25–34 years old	14%	14%	18%
% Female	51%	51%	53%
% Male	49%	49%	46%
% Democrat *	37%	57%	39%
% Independent *	26%	24%	28%
% Republican *	31%	20%	27%
% White/Caucasian	71%	74%	74%
% Black/African American	14%	14%	16%

**Table 2:** Participants’ likelihood of purchasing fur products.

Item	Overall	Connecticut	Massachusetts	New Jersey	New York
Purchase new clothing containing fur from animals raised on fur farms	29.4%	25.1%	29.8%	30.6%	32.2%
Purchase used/vintage clothing containing fur from animals raised on fur farms	31.5%	27.7%	32.2%	34.0%	32.3%

**Table 3:** Respondents’ agreement with a variety of belief statements related to fur.

Statement	Overall	Connecticut	Massachusetts	New Jersey	New York
	% Agree	% Agree	% Agree	% Agree	% Agree
Wearing fur is a symbol of wealth/social status	41.5	39.1	38.3	44.2	44.2
Buying fur is a right all people should have	42.0	39.1	41.5	45.0	42.4
Faux/fake fur is bad for the environment	26.3	22.5	26.6	25.8	30.4
Faux/fake fur is less fashionable/desirable	31.8	27.5	32.1	31.4	36.3
Used/vintage fur is more acceptable than new fur	43.6	42.5	46.0	44.4	41.7
The environmental impact of faux fur has improved	37.5	34.4	37.1	40.0	38.7
Fur farming can spread disease to humans	35.6	32.2	35.5	37.0	37.7
Faux/fake fur can suitably replace real fur	62.2	61.1	61.7	65.2	61.1
Gov’t shouldn’t regulate fur purchases	43.3	40.3	47.2	42.6	42.9
Fur farming is bad for the environment	41.3	42.9	40.5	42.2	39.7
Fur farming is cruel/inhumane to animals	60.7	64.2	59.7	62.4	56.5
U.S. fur farms are regulated for welfare standards	39.8	34.2	38.3	42.6	44.2

**Table 4:** Average treatment effect (ATE) of message condition on support for legislation banning the sale of fur from any animal, calculated from targeted maximum likelihood estimation (TMLE) models adjusting for social–psychological and demographic covariates.

Message Condition	ATE	95% CI (Lower)	95% CI (Upper)	*p* Value
Environmental	0.228	0.057	0.397	0.000
Animal welfare	0.256	0.078	0.434	0.005
Public health	0.236	0.064	0.408	0.007
Social norms	0.253	0.086	0.419	0.003
Economics/international	0.046	–0.123	0.216	0.593
Fur alternatives	0.101	–0.083	0.286	0.280

Note: ATE (average treatment effect): How much each message increased support for the fur ban legislation, measured on a scale where higher positive numbers mean greater increase in support.

**Table 5:** Average treatment effect of message condition on support for legislation. banning the sale of fur from animals raised in commercial fur farms, calculated from targeted maximum likelihood estimation (TMLE) models adjusting for social–psychological and demographic covariates.

Message Condition	ATE	95% CI (Lower)	95% CI (Upper)	*p* Value
Environmental	0.227	0.057	0.397	0.009
Animal welfare	0.256	0.078	0.434	0.005
Public health	0.236	0.064	0.408	0.007
Social norms	0.252	0.086	0.419	0.003
Economics/international	0.046	–0.123	0.216	0.593
Fur alternatives	0.101	–0.083	0.286	0.280

Note: ATE (average treatment effect): How much each message increased support for the fur ban legislation, measured on a scale where higher positive numbers mean greater increase in support.

## Data Availability

The original contributions presented in this study are included in the article. Further inquiries can be directed to the corresponding author.
